# Case Report: Prenatal Diagnosis of Postaxial Polydactyly With Bi-Allelic Variants in Smoothened (SMO)

**DOI:** 10.3389/fgene.2022.887082

**Published:** 2022-06-22

**Authors:** Lihong Fan, Pengzhen Jin, Yeqing Qian, Guosong Shen, Xueping Shen, Minyue Dong

**Affiliations:** ^1^ Center of Prenatal Diagnosis, Huzhou Maternity & Child Health Care Hospital, Huzhou, China; ^2^ Women’s Hospital, School of Medicine Zhejiang University, Hangzhou, China; ^3^ Key Laboratory of Reproductive Genetics (Zhejiang University), Ministry of Education, Hangzhou, China

**Keywords:** postaxial polydactyly, whole-exome sequencing, bi-allelic variants, smoothened, genetic counseling

## Abstract

Postaxial polydactyly is a common congenital malformation which involves complex genetic factors. This retrospective study analyzed the cytogenetic and molecular results of a Chinese fetus diagnosed with postaxial polydactyly of all four limbs. Fetal karyotyping and chromosomal microarray analysis (CMA) did not find any abnormality while trio whole-exome sequencing (trio-WES) identified bi-allelic variants in smoothened (*SMO*) and (NM_005631.5: c.1219C > G, NP_005622.1: p. Pro407Ala, and NM_005631.5: c.1619C > T, NP_005622.1: p. Ala540Val). Sanger sequencing validated these variants. The mutations are highly conserved across multiple species. In-depth bioinformatics analysis and familial co-segregation implied the compound heterozygous variants as the likely cause of postaxial polydactyly in this fetus. Our findings provided the basis for genetic counseling and will contribute to a better understanding of the complex genetic mechanism that underlies postaxial polydactyly.

## Introduction

**FIGURE 1:**
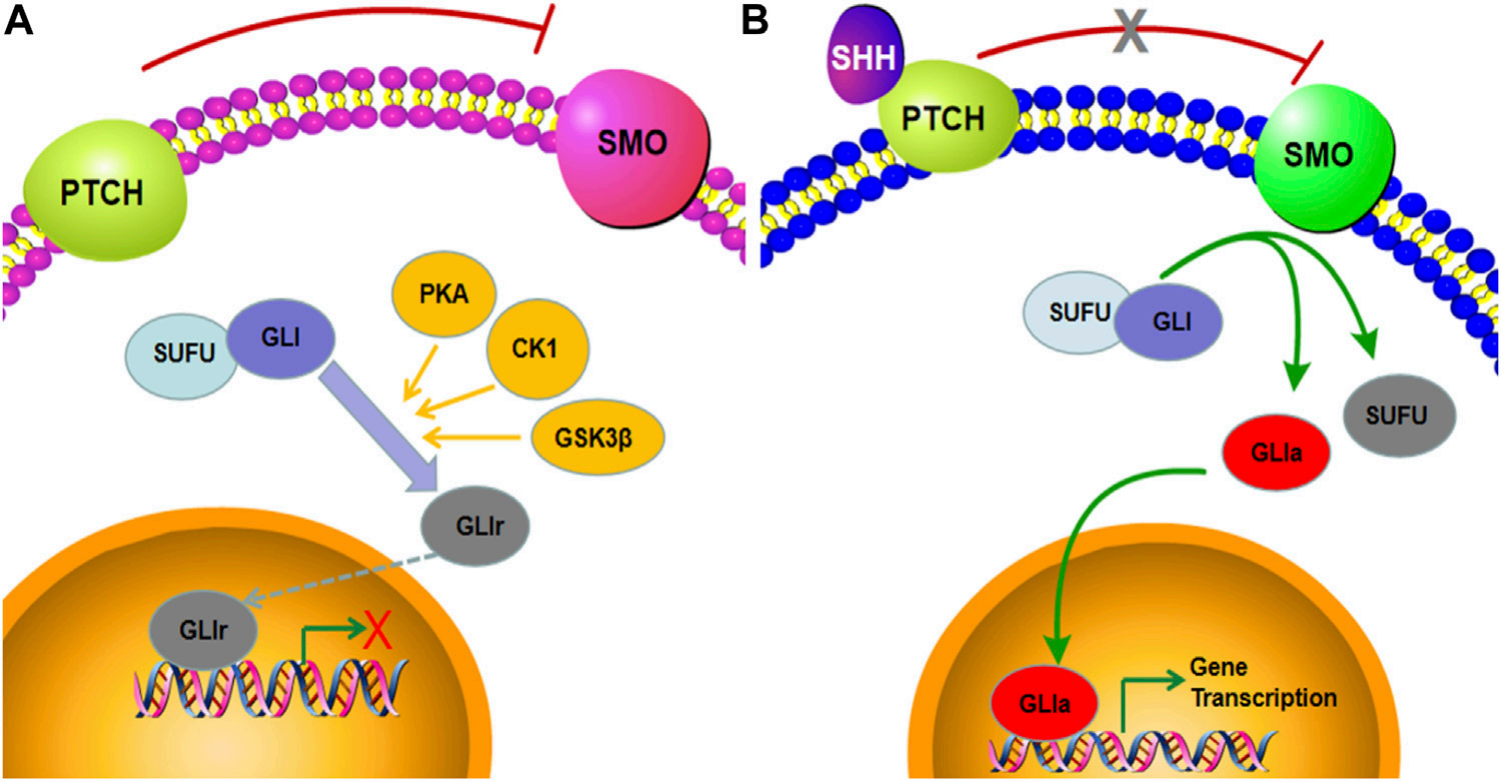
Graphical representation of active and inactive SHH signaling pathways. **(A)** When SHH is absent, the full-length GLI is phosphorylated by PKA, CK1, and GSK3β and proteolytic cleavaged into the GLI repressor, which subsequently suppresses the expression of SHH target genes. **(B)** In the presence of the SHH ligand, SMO inhibits the sequestration by SUFU and phosphorylation by PKA, CK1, and GSK3β, leading to the formation of the GLI activator and ultimately to induction of target gene transcription.

Postaxial polydactyly (PAP), characterized by an extra digit at the fifth finger or toe, is one of the most common congenital malformations (Umair et al., 2018). It can be a marker of a wide variety of neurological and systemic abnormities and usually occurs in syndromic types (Verma and El-Harouni, 2015; Chandra et al., 2017). Limb growth in vertebrates is regulated by a complex network of intercellular communication and gene expression. The Sonic hedgehog protein (SHH), one of three mammalian hedgehog (HH) proteins, is expressed in the zone of polarizing activity (ZPA) of the limb bud and those of the notochord and floor plate in the neural tube (Riddle et al., 1993; Johnson et al., 1994; Jessell, 2000; Xavier et al., 2016). There is compelling evidence that the SHH signaling pathway plays an important role in regulating the patterning and growth of the developing limb (Hill et al., 2003; Tickle and Barker, 2013; Chinnaiya et al., 2014; Choudhry et al., 2014). The major components of the SHH pathway include smoothened (SMO), PTCH, and GLI transcription factors. In the absence of ligand binding of SHH, PTCH binds to SMO and inhibits its activity. Here, SUFU binds to the GLI transcription factors, which are subsequently phosphorylated by PKA, CK1, and GSK3β and degraded by the proteasome. Under this condition, GLI is converted to GLIr with the C-terminal domain truncated and enters the nucleus, inhibiting the transcription of downstream target genes (Figure 1A). When SHH is present, it binds to the extracellular domain of PTCH and releases its repressive effects on SMO. Freed of PTCH1-mediated suppression, SMO relieves the sequestration by SUFU and phosphorylation from PKA, CK1, and GSK3β, thus stabilizing the GLI proteins in their full-length transcriptional activator form. The activated GLI (GLIa) translocates into the nucleus and promotes the transcription of SHH target genes (Figure 1B) (Briscoe and Thérond, 2013; Niida et al., 2021; Sigafoos et al., 2021). Here, we observed that the SHH signaling pathway is a highly regulated cascade of extracellular ligands, receptor proteins, cytoplasmic signaling molecules, transcription factors, coregulators, and target genes. Mutations in *SHH*, *PTCH*, *SMO*, and *GLI* can lead to the downregulation of the SHH pathway and ultimately to malformations (Johnson et al., 2014; Le et al., 2020; Yi et al., 2020). Most studies on PAP-related mutations have focused on *GLI*, while few studies, on *SMO* (Zou et al., 2019; Cao et al., 2020; Xiang et al., 2020; Patel et al., 2021)*.* To the best of our knowledge, no previous study on prenatal diagnosis of *SMO* mutations has been available so far. The present study reported novel bi-allelic variants in *SMO* likely causing the PAP in a fetus.

## Patients and Methods

### Case Presentation

**FIGURE 2:**
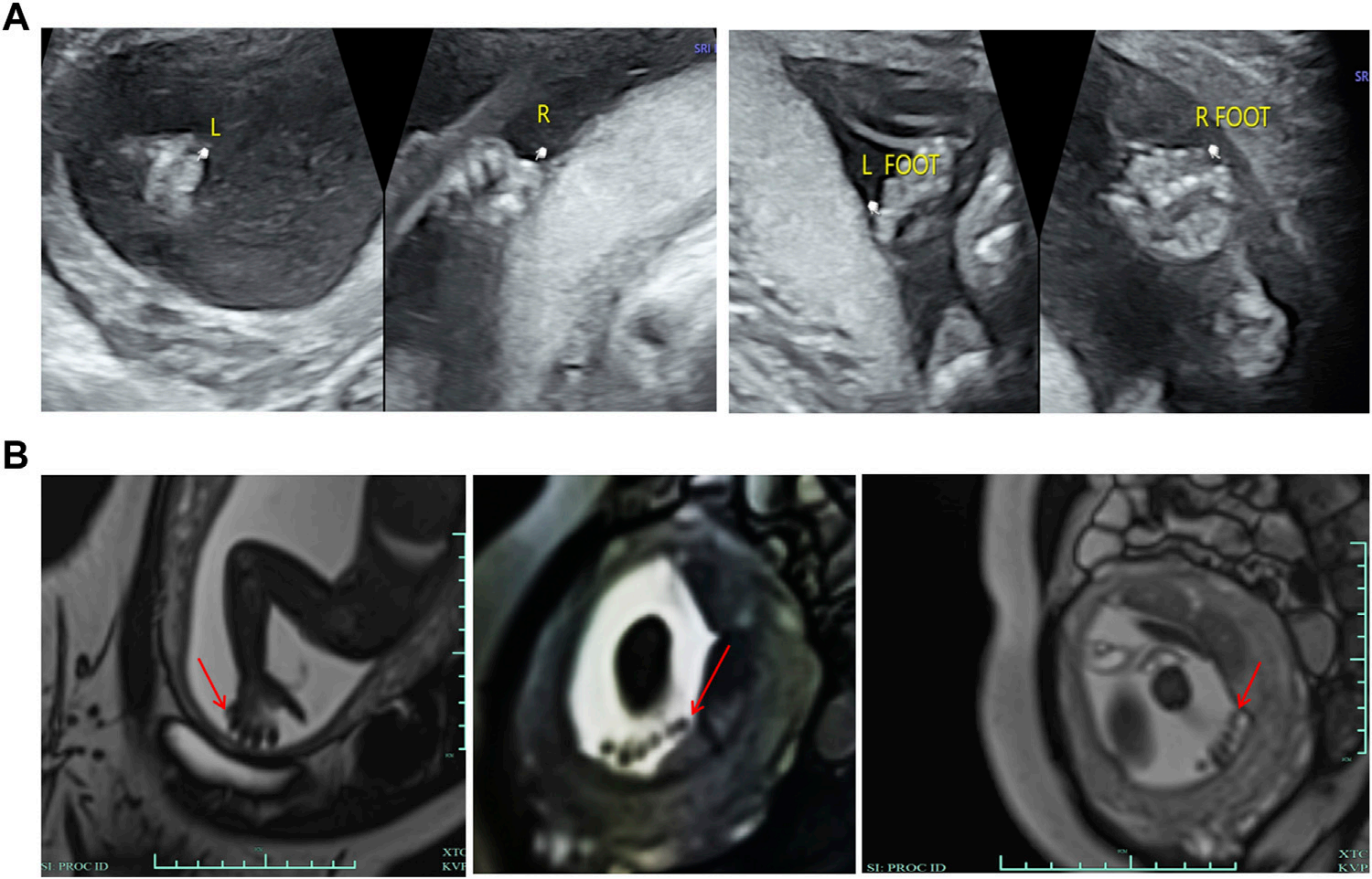
Clinical features of the fetus. **(A)** Ultrasonography showed that there were echoes of the sixth toe on the lateral side of the little toe of both feet and the lateral side of the little finger of both hands, with a size of about 0.6*0.4 cm, and no obvious bony structure in them. **(B)** MRI showed a finger-like signal shadow on the outside of the right little finger, while the number of fingers on the left hand was not clear due to the position of the fetus. Six toe-like signal shadows were seen on both feet.

A 29-year-old primigravida woman was referred to our center for further evaluation at 23^+2^ weeks of gestation. First-trimester screening and second-trimester screening for Down syndrome indicated a low risk. Routine prenatal ultrasound scans (US) at 22^+1^ weeks of gestation suggested the PAP of all four limbs (Figure 2A). MRI scans subsequently confirmed the result of fetal PAP (Figure 2B). The mother denied being exposed to teratogenic agents or irradiation or using nicotine or alcohol during the pregnancy. No family history of neurological disease or congenital malformations was recorded.

The current investigation was approved by the Ethics Committee of Women’s Hospital, School of Medicine Zhejiang University. All participants provided their written informed consent.

### Amniocentesis

Amniocentesis was performed aseptically under the guidance of ultrasonography at 23^+2^ weeks of gestation. Thirty milliliters of amniotic fluid were obtained, of which 15 mL was for cell culture, while the remaining 15 mL was for DNA extraction.

### Karyotype Analysis

Karyotype analysis included the culture of amniocytes in appropriate culture media and G-banded karyotyping at the 320–400 band level.

### Chromosomal Microarray Analysis

Genomic DNA was extracted from the amniotic fluid and the peripheral blood of the parents. Then, fetal DNA was analyzed using the CytoScan™ HD whole-genome SNP array (Affymetrix, United States), and data were analyzed by Chromosome Analysis Suite 4.2, according to manufacturers’ instructions.

### Trio Whole-Exome Sequencing and Sanger Sequencing

Genomic DNA extracted from the amniotic fluid and the peripheral blood of the parents was used for Trio-WES. Exome capture was performed using the SureSelect Human All Exon V4 kit (Agilent Technologies, Santa Clara, CA, United States), followed by sequencing using an Illumina HiSeq2500 system (Illumina, San Diego, CA, United States). After sequencing and filtering out low-quality reads, high-quality reads were compared to the GRCh37/hg19 reference human genome using Sentieon BWA (Sentieon, United States) with the MEM align method, and only the variants located in the coding sequence or splice site regions would be retained. Variant calling was performed using the Genome Analysis Tool Kit (GATK v4.0). Then, the candidate variants, including single-nucleotide variants (SNVs) and indels, were filtered by frequencies on specific databases, including the Human Gene Mutation Database (HGMD), ClinVar database, 1000 Genomes Project, Exome Aggregation Consortium (ExAC), Exome Sequencing Project 6500 (ESP6500), database of single-nucleotide polymorphisms (dbSNP), and Genome Aggregation Database (gnomAD). Mutation sites, known as polymorphic sites, were excluded, and the variants with allele frequency≤1% were retained. We used PolyPhen2, SIFT, Condel, MutationTaster, and phyloP to predict the effect of variants. The interpretation of sequence variants was performed according to the American College of Medical Genetics and Genomics (ACMG) (Richards et al., 2015). Online Clustal Omega (https://www.ebi.ac.uk/Tools/msa/clustalo/) was used to analyze the evolutionarily conserved sequences among species (humans, chimpanzees, macaques, mice, rats, *Sus scrofa*, horses, and *Bos taurus*). Finally, the identified variants were confirmed with Sanger sequencing. Sequences containing the two potential variants were amplified. The primer sequences were designed by Primer3 (http://primer3.ut.ee/), and all the primers are shown in Supplementary Table S1. Then, the PCR products were sequenced on the ABI 3500DX, followed by analysis by DNASTAR 5.0 software.

## Results

**FIGURE 3:**
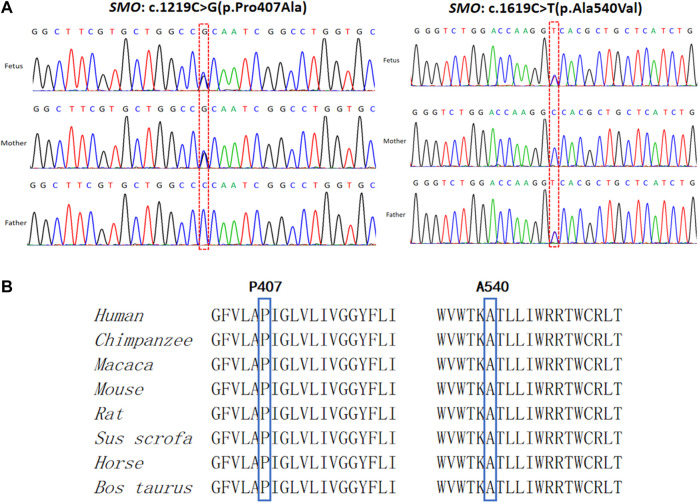
Genetic variations identified in this case. **(A)** Two variants detected in the fetus and parents. **(B)** Conservation of the mutated amino acids (Pro407 and Ala540) across different species.

CMA and karyotype analysis did not reveal any abnormalities. However, the trio-WES study identified a compound heterozygote in *SMO* (chr7:128846383, NM_005631.5: c.1219C > G, NP_005622.1: p. Pro407Ala; chr7:128850356, NM_005631.5: c.1619C > T, NP_005622.1: p. Ala540Val) inherited from the mother and father, respectively. Sanger sequencing confirmed the variants and showed that the two compound heterozygous mutations in *SMO* co-segregate with the disorder in this family (Figure 3A). The two mutations were absent in the 1000 Genomes, ESP6500, dbSNP144, and ExAC databases, and only the mutation c.1619C > T had a low allele frequency (0.00001989, all heterozygote) observed in gnomAD. The alignment of the amino acid sequences indicates that the two mutant residues were 100% conserved among many species (Figure 3B). Moreover, both variants were predicted to be pathogenic by the SIFT, PROVEAN, and MutationTaster tools (Table 1). Based on the consistency of genotype–phenotype correlation and the effect of the mutations, we speculated that the compound heterozygous mutations in *SMO* might be responsible for the limb abnormalities in this Chinese family.

**TABLE 1:** *In silico* analysis of the *SMO* variants c.1219C > G and c.1619C > T.

Variant	MutationTaster	PROVEAN	SIFT
c.1219C > G	Disease-causing	Deleterious	Damaging
c.1619C > T	Disease-causing	Deleterious	Damaging

Finally, the couple chose to terminate the pregnancy after carefully considering the abnormal ultrasonography findings and trio-WES results.

## Discussion

The genetic mechanism underlying polydactyly is highly complex. The SHH pathway is the essential evolutionarily conserved pathway related to the growth and patterning of the limbs (Patterson et al., 2009; Rahi and Mehan, 2020). SMO, a seven-transmembrane, encoded by *SMO*, is required for active SHH signaling (Zhang et al., 2001; Le et al., 2020). A previous study showed that removing *SMO* from the apical ectodermal ridge resulted in the loss of functional SHH signaling, ultimately leading to disruption of digit patterns and the formation of additional postaxial cartilage contraction (Bouldin et al., 2010).

In the present study, we identified bi-allelic variants in *SMO*, c.1219C > G (p. Pro407Ala), and c.1619C > T (p. Ala540Val) in a Chinese family. The second variant was previously reported in a Han Chinese boy, also in a state of compound heterozygosity (Zhang et al., 2019). It was predicted that this mutation affects the signaling ability of SMO as the alanine residue at position 540 is quite close to the seventh TM domain, which is considered to be the key site for signaling transduction. In addition, the previous study also performed a gene expression analysis and showed a significant decrease in the *SMO* expression in the patient compared with healthy controls. Back to our study, although the mRNA and protein level in the fetus cannot be detected due to the unavailability of the fetus’ sample, the pathogenic effect of Pro407Ala and Ala540Val substitution in SMO may be supported by the following points: 1) both loci were quite conserved among different species; 2) *in silico* bioinformatics applications predicted that both mutations are deleterious; 3) *SMO* belongs to recessive genes, and the two mutations co-segregated with the disorder in the family; 4) no other pathogenic variants were detected in the known polydactyly or limb development genes, such as *SHH*, *PTCH*, and *GLI*; and 5) the phenotype of PAP found in the fetus could be explained by *SMO* defects.

We searched the PubMed database to find relevant literature on *SMO* mutations. As a result, we found that the previous studies on *SMO* were limited and focused on activating somatic mutations that caused diseases such as sporadic basal cell carcinoma (BCC), medulloblastoma (MB), and Curry–Jones syndrome (CRJS) (Kool et al., 2014; Twigg et al., 2016; Bisceglia et al., 2020). Germline mutations, leading to developmental disorders, have not been reported until recent years. According to the previous reports, there were, in total, 10 patients with a series of congenital developmental abnormalities caused by *SMO* mutations (Rubino et al., 2018; Zhang et al., 2019; Le et al., 2020; Green et al., 2022). The fact that all the patients had compound heterozygous variants, while their parents were in a heterozygous state and had no associated phenotype, confirmed that *SMO* belongs to recessive genes. PAP can be observed in nine out of 10 patients, and 100% (9/9) of the patients with PAP also had other symptoms, such as thalamic hamartoma, cystic epilepsy, atrioventricular septal defect (AVSD), and aganglionosis. The most severe case was one who presented AVSD and died at 3 months of age (Le et al., 2020). In addition, all nine cases mentioned previously were diagnosed after birth, and our report on the fetus is to date the only prenatal genetic diagnosis of *SMO* mutations. We speculated that PAP may be the earliest prenatal manifestation and that the fetus may present other related symptoms after birth.

As one of the most common congenital malformations, PAP often appears as a key feature of malformation syndromes, such as Meckel–Gruber syndrome (Turkyilmaz et al., 2021), Bardet–Biedl syndrome (Ullah et al., 2017), and Pallister–Hall syndrome (Yang et al., 2021). According to previous studies, about 8% of bilateral PAP cases are related to a set of other congenital syndromic defects. As is well known, many syndromes have postnatal features that cannot be detected prenatally on ultrasound scans. The fetal phenotype is useful but sometimes limited source of information for the diagnosis of many Mendelian diseases. In prenatal cases with a less severe phenotype or an isolated anomaly, it is always quite difficult for parents to make a decision on continuing or terminating the pregnancy. We believe that prenatal WES is particularly valuable in detecting genetic variants of certain genes that may be critical to human development and helps us predict the postnatal phenotypes associated with the detected genes.

The most important limitation to our study lies in the fact that there is a lack of postpartum assessment and validation of associated malformations, such as thalamic hamartoma, cystic epilepsy, atrioventricular septal defect, and aganglionosis, due to our failure to get the permission for an autopsy. Therefore, it is still uncertain whether the fetus’ polydactyly was isolated or syndromic, although no abnormality in organs such as the heart and the brain was indicated by multiple prenatal ultrasounds and MRI. Given the very scarce reporting of occasional mutations, more studies in patients with related phenotypes are urgently needed to describe a full spectrum of *SMO* mutations. Detailed functional experiments are also needed to confirm how these mutations work.

In conclusion, we identified a novel compound heterozygous mutation (c.1219C > G, p. Pro407Ala and c.1619C > T, p. Ala540Val) in the gene *SMO.* There is convincing evidence, although no definite proof, that this mutation is causative for the PAP in the present family. The genetic diagnosis provided evidence for assessing the risk of recurrence and was invaluable for genetic counseling of the couple contemplating future pregnancies.

## Data Availability

The datasets for this article are not publicly available due to concerns regarding participant/patient anonymity. Requests to access the datasets should be directed to the corresponding author.
