# Supplementary material for: Case Report: Prenatal Diagnosis of Postaxial Polydactyly With Bi-Allelic Variants in Smoothened (SMO)
Source: Front Genet. 2022 Jun 22;13:887082. doi: 10.3389/fgene.2022.887082 (PMC9257524; doi:10.3389/fgene.2022.887082)
Supplement: Supplementary file 1 [file Table1.DOC]

**Supplementary Table S1. Primer sequences used to amplify *SMO* genomic fragments**

| **Name of Prime** | **Primer sequence (5’→3’)** |
| --- | --- |
| Exon6-*SMO*-F | GGTATAGTGACTGGTAGGAACG |
| Exon6-*SMO*-R | AAGTGCTCACGGTGTTTCGTCT |
| Exon9-*SMO*-F | TCACCTGTCTACGTTCCCTCAC |
| Exon9-*SMO*-R | ACTGACCAAGGCTGTGCTAGAG |
